# Prevalence of Dental Caries in Arabian Children Attending King Fahad Armed Forces Hospital, Jeddah: A Pilot Study

**DOI:** 10.7759/cureus.89477

**Published:** 2025-08-06

**Authors:** Abdulaziz Alsaif, Mohammed Badi Albadi, Rashad Nabeel Nageeb, Reem M Azad Allarakia, Khalid M Alzahrani

**Affiliations:** 1 Pediatric Dentistry, Prince Abdulrahman Advanced Dental Institute, Riyadh, SAU; 2 Dentistry, Saudi Royal Guard Medical Services, Riyadh, SAU; 3 Dentistry, Alhada Armed Forces Hospital, Al Hada, SAU; 4 Pediatric Dentistry, King Fahad Armed Forces Hospital, Jeddah, SAU; 5 Restorative Dentistry, Prince Abdulrahman Advanced Dental Institute, Riyadh, SAU

**Keywords:** arabian children, dental caries, dental visit, primary dentition, tooth brushing

## Abstract

This study aimed to identify the prevalence of dental caries in children and the impact of associated risk factors. This cross-sectional study was performed on children attending the dental clinic at King Fahad Armed Forces Hospital in Jeddah, Saudi Arabia. The American Academy of Pediatric Dentistry criteria were used for the diagnosis of early childhood caries. Only Saudi children aged three to 12 years whose parents had given consent and who were attending dental clinics at King Fahad Armed Forces Hospital, Jeddah, were involved in the study and underwent clinical examinations. Non-Saudi nationals and uncooperative children under the age of three years are excluded from the study. The statistics were performed using SPSS version 23 (Armonk, NY: IBM Corp.), considering a p-value of less than 0.05 as significant. A high prevalence of 95% (96) of dental caries was evident in the present study. This study found that dental visits and diet had an impact on dental caries status, while gender, age, family position, and oral hygiene practices did not show any impact.

## Introduction

Dental caries continues to be a significant public health issue and is the leading cause of many childhood impairments [1]. Children with caries in their primary dentition are three times more likely to acquire caries in their permanent teeth, making severe early childhood caries extremely difficult to control [2]. The patient's caries risk refers to the likelihood that a child may develop new caries lesions (active white spots, non-cavitated approximal lesions, and cavitated lesions) in the near future. A crucial individual caries risk assessment, and a personalized treatment plan based on the assessment data, is essential for managing dental caries [3]. Dental caries continues to be the most frequent oral disease in children, especially in developing countries. Despite widespread efforts to curb its spread and recent improvements in public awareness of oral and dental health, the problem is still widely prevalent. Dental caries is a worldwide public health concern. In children, dental caries is five times more prevalent than asthma and seven times more common than allergic rhinitis. In addition to malnutrition, obesity, and allergies, dental caries presents a major obstacle throughout the developmental stages of childhood [4-6]. The causes of tooth decay are multifaceted. While host, microflora, substrate, and time are the four most important elements, there are many other factors that contribute to dental caries but are not evident in clinical examinations [5]. It is believed that these factors heighten the likelihood of developing dental caries [6]. Risk factors are not direct causes of disease, but they do play a substantial role in disease development and are thus significant. Risk factors can explain the disease management imbalance in pathological conditions that occur following the onset of clinical disease [7-9]. Untreated dental caries can negatively impact a child's quality of life in several ways, including through pain and discomfort that can lead to disfigurement, cognitive development, acute and chronic infections, and alterations in eating and sleeping habits; hospitalization, costly treatment, missed school days, and reduced learning ability [10-12].

Most of the time, it is possible to prevent cavities in teeth. To better understand the patient's cariogenic profile, it is crucial to first identify the caries risk. To implement effective protocols and preventive measures, we require detailed knowledge of the population-level risk factors for caries. Regardless of how much they try, children can never become truly self-reliant; they will always need a nurturing adult figure like a mother. There is an increased risk of dental caries in children due to environmental factors, particularly the maternal environment [13]. Mothers are instrumental in developing their children's personalities [14]. Many reports have shown a correlation between a mother's socioeconomic position, health attitudes, and oral health habits and her children's oral health [15,16]. Mothers influence their children's dental health as they provide comfort and security. Maternal factors strongly influence infant caries risk. The discussion above makes it clear that it is unrealistic to separate paternal factors from the identification of caries risk factors in children. They should explore the demographic characteristics, mother's attitude and knowledge, and clinical condition as potential risk factors for caries. Cariogram, Caries Management by Risk Assessment (CAMBRA), Caries Risk Assessment Tool (CAT), PreVisor, and the National University of Singapore Caries Risk Assessment Tool (NUS-CRA) are some of the methods available for Caries Risk Assessment (CRA) [17-20]. The American Academy of Pediatric Dentistry pioneered the CAT. Evidence suggests that multivariate models are superior to those with only one predictor, and an effective model is one that considers the most relevant factors while remaining simple for patients and caregivers to use and understand, especially when working with children. The model considers one's clinical status, surrounding environment, and general health when determining an appropriate level of risk. Then, children are put into one of three cavity risk categories [21]. The practitioner must describe each patient's caries risk status to provide the most effective preventative measure. The idea of a personalized caries risk assessment is to tailor each child's diagnostic testing, preventative measures, and restorative care to their individual needs [22]. The CAT has the potential to play a pivotal role in the evaluation and prevention of dental caries, especially in light of the increasing prevalence of caries in Saudi Arabian children [23,24]. There is no recent data on the caries risk status of children living in Jeddah, and previous studies were done a decade ago. Researchers have used the American Academy of Pediatric Dentistry as a tool to assess caries risk and measure the caries risk status [25,26]. Various studies [2,4,6,8,10] published in Saudi Arabia focus on different aspects of dental health and related risk factors. However, there is a need to identify the associated risk factors of dental caries. Henceforth, the objective of the study was to determine the caries prevalence and associated risk factors.

## Materials and methods

The Medical Services Department for Armed Forces, Scientific Research Center, Research Ethics Committee granted #2023-11 ethical approval for this study. Children aged three to 12 years participated in a cross-sectional study at the dental clinic at King Fahad Armed Forces Hospital in Jeddah, Saudi Arabia. In this study, we assessed children aged 3-12 years who were attending dental clinics using a convenience sampling approach. Informed consent was taken from the parents of the children after explaining the need for and the benefits of this research. The study was conducted during the period from March 2023 to June 2023. A minimum sample size was determined considering the prevalence of caries in schoolchildren from recent research conducted in Jeddah by Vanka et al. [27]. The participating children were measured using the American Academy of Pediatric Dentistry (AAPD), a gold standard caries risk assessment instrument, which evaluates the risk based on the clinical status of the child, environmental factors, and general health. Children were assessed for the following conditions: recent incipient or cavitated caries lesions, tooth loss within the past 24 months as a result of caries or restoration, the existence of an appliance in the mouth, and children having special healthcare needs.

Questionnaire

The standard caries risk assessment tool classifies children as low, moderate, and high risk for future lesions. The questionnaire included blinded names, risk and protective factors for caries, general health, and a clinical examination (table in appendix). The children visited the dentist for a visual inspection using an operating light, a dental mirror, and a triple syringe while they sat in a dental chair. The caries risk indicators encompass variables like dental plaque, which either directly contribute to caries or serve as reliable predictors of the illness, such as the time, frequency, and duration of sugar consumption. Some indicators of caries risk activities could include the appearance of new incipient or cavitated caries, tooth loss due to caries, or restorations made within the last 24 months. Children who receive topical fluoride from a health professional and brush their teeth daily with fluoridated dentifrice reduce the caries risk. The clinical examination's visual component also included whether the mother had caries lesions in the past six to twenty-four months. For ease and convenience, we only considered professional fluoride application as fluoride exposure and excluded other times of fluoride exposure. The question "established record of the patient receiving regular dental treatment in a dental clinic" was replaced by "regular brushing."

Inclusion and exclusion criteria

All children aged three to 12 years of Saudi nationality, whose parents provided informed consent and who underwent clinical examinations at dental clinics in Jeddah, were included in the study. Uncooperative children, below three years of age, or of non-Saudi nationality were excluded.

Calibration and statistical analysis

A pilot study was conducted on 20-25 children, and to avoid bias, a single examiner was involved in the data collection with an excellent kappa value (0.8). The data from the pro forma form was entered into a Microsoft Excel sheet (Redmond, WA: Microsoft Corp.) and transferred to statistical software (SPSS version 23; Armonk, NY: IBM Corp.) for statistical analysis. An independent biostatistician analyzed the data, which could potentially reduce the assessment bias. Categorical variables were presented using percentages and frequencies. Possible associations between categorical variables were assessed using Pearson’s chi-square test. The multivariate regression analysis was performed to establish the role of dental caries scores on gender, number of children in the family, child's first dental visit, child's sugary diet, age of the child, and visible plaque, with female (gender), one to three (number in the family), never (first dental visit), no (sugar foods), three to five years (age of the child), and no (visible plaque) used as a reference.

## Results

A total of 101 children participated in the study with a mean of 7.3±2.4 years. Among them, 47% (48) were males with an average age of 6.9±2.4 years, while 53% (53) were females with an average age of 7.5±2.3 years. Among the total study population, 54.5% (55) had visible plaque; among them, 51% (28) were female and 49% (27) were male. Thirty-seven (36.6%) children who participated in the study exhibited enamel defects, with 46% (17) being female and 54% (20) being male. Fifty-six (55.4%) children in the study were observed with non-cavitated carious lesions; among them, 28 (50%) were males and 28 (50%) were females. Eighty-five (84.2%) children observed with visible carious lesions, among them, 40 (53%) were males, while 45 (47%) were females. Sixty-four (63.4%) study population had recent restorations or missing teeth due to caries; among them, 37 (58%) were females and 27 (42%) were males. Table 1 summarizes the descriptive details of the age and oral health status of the children who participated in the study.

**Table 1:** Descriptive details of the age and oral health status of the children participated in the study. Dmft: decayed, missing, and filled teeth in permanent dentition

Dmft	Male (n=48), n (%)	Female (n=53), n (%)	Total (n=101), n (%)
Age of the child, mean (SD)	6.9 (2.4)	7.5 (2.3)	7.3 (2.4)
The child has visible plaque on the teeth	27 (56.3)	28 (52.8)	55 (54.5)
The child presents with dental enamel defects	20 (41.7)	17 (32.1)	37 (36.6)
The child has non-cavitated (incipient/white spot) caries lesions	28 (58.3)	28 (52.8)	56 (55.4)
The child has visible caries lesions	45 (93.8)	40 (75.5)	85 (84.2)
The child has recent restorations or missing teeth due to caries	27 (56.3)	37 (69.8)	64 (63.4)

The study's findings revealed a high prevalence of dental caries, accounting for 95% (96). The children in the study ranged in age from three to 12 years, with a mean of 7.5 years. Within their families, children are typically the first or second in line of birth. Of the children who visit the dental clinic, 22.8% (23) have visited previously, while 77.2% (78) were making their first visit. The comparison of sugar intake of children and their caries statuses was found to be non-significant (p=1, χ²=0.023). Additionally, it was shown that 64.4% (65) of children ingest sugars (sweets and juices) more than three times a day in between meals, whereas only 35.6% (36) of children don't. This study found the comparison between children's sugar intake and their caries statuses to be significant (p=0.001, χ²=9.49). There is variation in children's tooth-brushing habits; 38.6% (39) of them brush twice a day, 34.7% (35) brush once a day, and 24.8% (25) do not brush at all. The comparison of brushing habits of children and their caries statuses was found to be non-significant (p=0.254; χ²=4.066). Enamel defects, carious lesions, and visible plaque were frequently found with varying frequencies; 45.5% (46) of children do not have visible plaque on their teeth, compared to 54.5% (55) of children with visible plaque. The study found a significant comparison between the visible plaque in children and their caries statuses (p=0.004; χ²=6.29). Of children, 36.6% (37) exhibit evidence of tooth enamel defects, whereas 63.4% do not. There are some variations in ages among guardians or caregivers, with an average age of about 41.6 years. Forty-eight (47%) guardians have a bachelor's degree, despite their diverse educational backgrounds. But only 5% (5) held master's degrees, compared to 39.6% (40) who merely had a high school diploma, indicating a wide range of educational backgrounds. The comparison of the education of parents with caries statuses was found to be significant with a p-value of 0.053 (χ²=12.45). These findings provide information for additional research and intervention techniques by estimating the participant demographics and dental health-related habits. There was no statistically significant correlation observed (p>0.05). The demographics of the study sample were summarized in Table 2.

**Table 2:** Demographic characteristics of the study sample.

Parameters	Caries free (n=5), n (%)	Caries (n=96), n (%)	Chi-square value	p-Value	Total (n=101), n (%)
Parental education					
Bachelor's degree	4 (80.0)	44 (45.8)	12.45	0.053	48 (47.5)
Beginning education	0 (0)	1 (1.0)			1 (1.0)
High school education	0 (0)	40 (41.7)			40 (39.6)
Higher diploma	0 (0)	4 (4.2)			4 (4)
Illiterate	0 (0)	1 (1.0)			1 (1.0)
Intermediate education	1 (20.0)	1 (1.0)			2 (2.0)
Master's degree	0 (0)	5 (5.2)			5 (5.0)
Gender of the child					
Male	0 (0)	48 (50.0)	4.64	0.029	48 (47.5)
Female	5 (100)	48 (50.0)			53 (52.5)
Arrangement of the child in the family					
1-3	4 (7)	51 (93)	4.338	0.740	55 (16.8)
4-5	1 (4)	24 (96)			25 (100)
>5	0 (0)	21 (100)			21 (100)
Is this your first visit to a dental clinic?					
Yes	1 (20.0)	22 (22.9)	0.023	1	23 (22.8)
No	4 (80.0)	74 (77.1)			78 (77.2)
Does the child eat sugar (sweets and juices) between meals more than three times a day?					
Yes	0 (0)	65 (67.7)	9.49	0.001	65 (64.4)
No	5 (100.0)	31 (32.3)			36 (35.6)
How many times does your child brush his teeth a day?					
Never	0 (0)	2 (2.1)	4.066	0.254	2 (2.0)
Once	1 (20.0)	34 (35.4)			35 (34.7)
Twice	4 (80.0)	35 (36.5)			39 (38.6)
Brushes his teeth irregularly	0 (0)	25 (26.0)			25 (24.8)
Age of the child					
3-5 years	1 (20.0)	27 (28.1)	0.157	0.683	28 (27.7)
Above 6 years	4 (80.0)	69 (71.9)			73 (72.3)
Child has visible plaque on teeth					
Yes	0 (0)	55 (57.3)	6.290	0.004	55 (54.5)
No	5 (100.0)	41 (42.7)			46 (45.5)

The multivariate regression analysis performed to establish the role of dental caries scores on gender, number of children in the family, child's first dental visit, child's sugary diet, age of the child, and visible plaque, whereas gender, number of children in the family, child's first dental visit, child's sugary diet, age of the child, and visible plaque were not found to impact the dental caries scores (p>0.005) (Table 3).

**Table 3:** The multivariate linear regression analysis used the association between demographic variables dental carious scores.

Parameters	Category	B	SE	OR	Test statistic	Significance
Gender of the child	Male	18.9	5801	>1	0.0	>0.05
	Female (reference group)	-	-	-	-	-
Arrangement of the child in the family	1-3 (reference group)	-	-	-	-	-
	4-5	0.453	1.14	>1	0.157	>0.05
	>5	18.47	12118.2	>1	0.000	>0.05
Is this your first visit to a dental clinic?	Yes	0.17	1.14	>1	0.00	<0.05
	No (reference group)	-	-	-	-	-
Does the child eat sugar (sweets and juices) between meals more than three times a day?	Yes	19.38	4985.324	>1	0.00	<0.05
	No (reference group)	-	-	-	-	-
Age of the child	3-5 years	0.44	1.14	>1	0.15	>0.05
	Above 6 years (reference group)	-	-	-	-	-
Child has visible plaque	Yes	19.0	5410.69	>1	0.00	>0.05
	No (reference group)	-	-	-	-	-

## Discussion

A recent systematic review found the dental caries proportion level ranged from 0.21 to 1.00 for primary teeth [28]. This information indicates that the prevalence of dental caries in Saudi Arabia is high. The authors reported that the quality of the methodology used in the included studies was neither appropriate nor standardized. However, the current data do not provide a complete assessment of dental caries across Saudi Arabia. The authors in the present study set out to quantify the frequency of early childhood caries (ECC) and its associated risk factors among preschoolers in Jeddah aged three to five years. To understand the diagnosis of dental caries in children, the American Academy of Pediatric Dentistry provided criteria used to diagnose ECC. The authors used the decayed, missing, and filled teeth (dmft) and decayed, missing, and filled surfaces (dmfs) indices, along with sociodemographic data collected via a questionnaire to determine the ECC status of the children who participated in the study. In developing the questionnaire, the authors referred to a previous restudy performed on risk variables. To ensure its validity and reliability, the researchers conducted a pilot study with a group unrelated to the research population. Eleven percent of the preschoolers in this research had ECC. Children aged three to five had caries in 54.5% of cases, 58% in four-year-olds, and 56.6% in five-year-olds.

By improving the oral healthcare delivery system, we can reduce the incidence of caries. A community- and school-wide effort is necessary to raise awareness and prevent the development of tooth decay in young children. Dentists and dental public health workers working together in community events can help decrease the prevalence of cavities and catch them early [29-32]. Furthermore, lifestyle choices, dietary patterns, social standing, and sociodemographic variables all play a role in the development of dental caries. Reduced sugar consumption, proper tooth brushing practices, and routine examinations can all help avoid cavities [29,32]. In Saudi Arabia, there has been evidence of a rise in the prevalence of caries in recent decades [33]. According to four studies published between 2008 and 2018, the prevalence of dental caries varied across Saudi Arabia, ranging from 49 to 91.3% in different areas [34-38]. Research in Jeddah has linked frequent dental checkups to an increased risk of ECC in children [27]. Fifty percent of people who took part in the survey said they've never been to the dentist. While it's pleasing to see that many parents are taking their kids to the dentist, there has to be a way to make sure those parents are aware of the importance of regular dental checkups, as dental caries affected 58% of the children who visited the dentist for checkups [39,40].

Previous studies have indicated a correlation between children's dental caries and their age [40], sex [41,42], parents' educational level [43], and socioeconomic status [44]. Nonetheless, there is much disagreement over the study's findings. Thus, in our study, there was no significant correlation between guardians' academic achievement and whether children exhibited evidence of visible caries, enamel defects, or non-cavitated incipient white spot caries lesions [45]. Moreover, we did not find any meaningful link between the children's gender and the presence of enamel defects, non-cavitated incipient white spot caries lesions, or visible caries. Azizi concluded that there is no correlation between socioeconomic background and dental health [46], despite Pakpour et al. finding a correlation between children's oral health status and factors such as age, sex, and family income in Iran [44,47]. These differences may be attributed to cultural and behavioral variations across regions, as research by Farsi has shown that cultural background and family values are associated with a lower incidence of dental cavities [48]. Furthermore, the researchers discovered that different social groups required distinct approaches to assist children in developing better dental hygiene practices [48]. Therefore, authorities need to know the precise status of children's dental caries and its related determinants in that particular region to establish strategies for promoting oral health in children from diverse places with varied cultures [49].

Farsi studied four- and five-year-olds and discovered that 61% of the four-year-olds and 67% of the five-year-olds had caries [48,50]. Al-Malik et al. discovered a 73% prevalence in a 2002 study of Jeddah children aged three to five years [49,51]. The prevalence was 61% among children aged three years, 73% among those aged four years, and 76% among those aged five years. For instance, 83% of Palestinians and 76% of Emiratis have high rates of ECC [52,53]. Still, the caries rate in the current work was 95%. We calculated the sample size using a previous systematic study that projected the dental caries prevalence among five- to seven-year-olds at 84%. Although the sample size was small, the children surveyed are likely representative of Saudi children who have primary dentition. This suggests a high incidence of dental caries in Saudi Arabia. According to earlier published research in the literature [14], the reported prevalence of caries in primary teeth ranges from 57.2% to 91.2% [6]. The results of this analysis indicate that, on the whole, the included studies' methodological quality is lacking [54].

Dental caries is a problem that lowers the quality of life for those who are affected and has a significant financial impact on people individually and on society as a whole [40]. Tooth decay can cause pain that interferes with speaking, eating, and attending school, which can hinder development and growth [29,30]. It is agreed in the literature that there is a very high prevalence of dental caries in children. Among the variables that contributed to the rise of dental caries were the increased use of sugary foods, inadequate oral hygiene practices, insufficient brushing habits, and low levels of awareness. Finlayson et al. found that certain cognitive, behavioral, and psychosocial traits of mothers were associated with the way their children brush their teeth [53,54]. Additionally, the dentist should emphasize the significance of a child's first dental visit at the age of one year. The next step is to encourage routine checkups beginning at a young age to reduce the prevalence of caries in youngsters. A public health concern is the prevalence of caries in youngsters. By establishing both immediate and distant objectives, one may steer clear of ECC. Remember that sick kids are more likely to see the dentist. This observation agrees with what Beil et al. and Vanka et al. found in their investigation [38,27].

Regular dental visits can improve oral health in children, leading to better oral hygiene, fewer dental problems, and control over sugary drinks [6,38]. Thomson et al. found that children who had visited had higher dmft scores compared to those who had never visited before [54]. This effect could be due to children who required treatment or expressed concerns about visiting the dentist. Researchers have also supported this concept, stating that visiting the dentist not only has a therapeutic component but also includes educational interventions that promote oral health. Pediatric dentists can help achieve this goal by providing therapeutic and educational benefits. The present study showed similar results compared to the published literature: the dental visit and sugary diet had a significant impact on the dental carious status of the study population. Tooth brushing habits significantly reduce the prevalence of dental caries. A study found that only 66.4% of participants brushed their teeth at least once, and 15.7% more than once [55]. However, another study found 81% of children brushed their teeth daily, with 61% brushing twice a day [6]. Contrarily, the present study reported that there was no impact of tooth brushing on dental carious status. Regular brushing resulted in better dmft scores for children, and excellent brushing habits were associated with a low incidence of dental caries. Overall, regular tooth brushing habits can significantly reduce dental caries.

The incidence of dental caries has been steadily declining in children in Jeddah who are three to five years old. Parents and children may have a higher level of knowledge, which could potentially explain this trend. One possible explanation for the decreasing trend in caries prevalence in Jeddah is the improved availability of dental care. The increase might be because there have been more dental institutions, hospitals, and clinics in the city. There is still a significant need to improve caries preventive methods, given that the prevalence of caries remains relatively high, despite a decreasing trend. Since this study was cross-sectional, all study-specific limitations should be taken into account. Therefore, to more precisely identify the variables influencing children's dental caries status, we recommend a better study design, especially a cohort study. Additionally, this study screened primary and permanent teeth for dental caries; however, it was not made clear whether the carious lesion was in a primary or permanent tooth. Given that the study is institutionally based, the number of patients in healthcare facilities may be larger than in the population, indicating the need for oral health promotion. Variations in the research population, period, and environment could potentially account for the high frequency of dental caries in this investigation. We recommend a community-based study for a more accurate assessment. Parental education cannot be performed with the logistic regression model since the illiterate is considered a reference. All the children attending the dental hospital at King Fahad Armed Forces Hospital in Jeddah for the first time have been involved. The sample size was very small in the study, which is a major limitation. The present study did not evaluate the socioeconomic status of the parents. This is also considered a potential limitation of the study. The study population was from an outpatient clinic at the university, which is also a limitation of the study due to selection bias. However, this study can be a reference for further studies.

## Conclusions

This study observed a high prevalence of dental caries in the children. The study found that dental visits and diet had an impact on dental caries status, while gender, age, family position, and oral hygiene practices did not show any impact. The study highlights the need for community-level interventions; integrating oral health promotion programs with other health services may have a significant impact and help address the community’s overall oral health issues. Dental caries prevention is a major concern, and oral hygiene promotion is essential.

## Appendices

**Table 4:** The questionnaire used in this study.

Questionnaire
Hospital ID:
Gender of parent: male/female
Age of the child (years)
Parents’ education level: father/mother
Bachelor's degree
Beginning education
High school education
Higher diploma
Illiterate
Intermediate education
Master's degree
Gender of the child
Male
Female
Birth order of the child in the family
1-3
4-5
>5
Is this your first visit to a dental clinic?
Yes
No
Does the child eat sugar (sweets and juices) between meals more than three times a day?
Yes
No
How many times does your child brush his teeth a day?
Never
Once
Twice
Brushes his teeth irregularly
Age of the child
3-5 years
Above 6 years
Dental chart
Permanent teeth
Upper Jaw: 18 17 16 15 14 13 12 11 | 21 22 23 24 25 26 27 28
Lower Jaw: 48 47 46 45 44 43 42 41 | 31 32 33 34 35 36 37 38
Primary (deciduous) teeth
Upper Jaw: 55 54 53 52 51 | 61 62 63 64 65
Lower Jaw: 85 84 83 82 81 | 71 72 73 74 75
The child has visible plaque on the teeth
The child presents with dental enamel defects
The child has non-cavitated (incipient/white spot) caries lesions
The child has visible caries lesions
The child has recent restorations or missing teeth due to caries
